# RBBP6 expressional effects on cell proliferation and apoptosis in breast cancer cell lines with distinct p53 statuses

**DOI:** 10.2147/CMAR.S169577

**Published:** 2018-09-10

**Authors:** Lesetja Raymond Motadi, Mashianoke Marcia Lekganyane, Pontsho Moela

**Affiliations:** 1 Department of Biochemistry, Faculty of Agriculture, Science and Technology, North-West University (Mafikeng Campus), Potchefstroom, South Africa, lesetja.motadi@nwu.ac.za; 2 Department of Genetics, Faculty of Science, University of Pretoria, Pretoria, South Africa

**Keywords:** breast cancer, p53, apoptosis, RBBP6

## Abstract

**Introduction:**

Breast cancer is the most common malignancy amongst women and has a higher incidence rate than lung cancer. Its tumor progression partially results from inactivation of p53 which is caused by overexpression of ubiquitous regulatory proteins possessing p53-binding domain. RBBP6 is regarded as one of the ubiquitous proteins because of its RING finger-like domain which enables it to possess E3 ligase activity. Thus, it has become a potential target in cancer treatment as it is highly expressed in various malignancies including cancer. However, it is not clearly defined whether the effect of RBBP6 on cell growth and apoptosis is cell line-dependent, more especially in breast cancer cell lines that have distinct p53 expression profiles. This study aims at evaluating the effects of RBBP6 on cell growth and apoptosis in breast cancer cell lines with different p53 expressions.

**Methods:**

Following the analysis at mRNA and protein levels in breast cancer tissue, RBBP6 expression was successfully manipulated using gene silencing and protein overexpression techniques in MCF-7 and MDA-MB-231 cell lines. The cells were co-treated with siRBBP6 and anticancer agents following apoptosis detection, which was confirmed by caspase 3/7 activity and quantification of apoptotic genes.

**Results:**

RBBP6 was overexpressed in breast cancer tissues that were classified as stages 3 and 4, while in stage 1, its expression was much lower. The MCF-7 cell line which expresses wild-type p53 was more sensitive to apoptosis induction than MDA-MB-231 which is a mutant p53-expressing cell line. These data suggest that RBBP6 silencing triggers significant levels of intrinsic apoptosis, and its overexpression appears to promote cell proliferation in wild-type p53-expressing MCF-7 cell line as opposed to MDA-MB-231 cells.

**Conclusion:**

The effect of RBBP6 on cell proliferation and apoptosis induction in breast cancer seems to be cell line-dependent based on p53 status.

## Introduction

Breast cancer remains a female-related health problem on a global scale, accounting for over a million newly estimated cases and the counts are still on the rise.^1^ Uncontrolledcell growth and metastasis are considered the hallmarks of not only breast tumorigenesis but also of most other cancers. These malignant transformations are due to mutations and/or inactivation of genes involved in the regulation of cell cycle and apoptosis.^1^,^2^ WithTP53 being the most common tumor suppressor gene, it has been found to be mutated in over 50% of most human cancer types.^3^ In breast cancer, the frequency of p53 mutations varies greatly between the heterogeneous subtypes, with basal-like breast cancers having the highest frequency whereas the luminal subtypes have been shown to generally express wild-type (wt) p53.^4^ Under normal cellular conditions, wt p53 levels are kept in check by MDM2 negative regulator; however, this event is cancer promoting during transformation. This is because MDM2 inhibits p53 transcriptional activity by facilitating its nuclear transport, thus triggering degradation via the ubiquitin proteasome pathway. A lot of research has been done in which the p53–MDM2 interaction has been successfully disrupted.^4^ Another extensively studied ubiquitous protein that has been shown to negatively regulate wt p53 is called E6 oncoprotein in cervical cancers since it possesses the E3 ligase activity, an important function that is needed during cancer development.^5^ RBBP6 is yet another suspected deregulator of wt p53 due to its E3 ligase activity as well as the presence of p53, DWNN and RING finger-like domains.^6^ However, the underlying mechanism in which RBBP6 negatively regulates wt p53 is currently unclear. In our previous study, we have shown that silencing RBBP6 led to wt p53 restoration that resulted in apoptosis induction.^7^ These observations prompted us to carry out a comparative study between a cell line that expresses wt p53 and that which expresses mutant (mt) p53. The aim of this manuscript was therefore to overexpress and silence RBBP6 gene expression in the mt p53-expressing MDA-MB-231 breast cancer cell line in comparison to wt p53-expressing MCF-7 and analyze its effects on cell proliferation and apoptosis.

## Materials and methods

### Materials

Breast cancer tissue sections were obtained from National Health Laboratory Service Department of Anatomical Pathology following classification by Dr J Murry (ethical approval number NWU00409-17-A9; North-West University). Human cancer cell lines MCF7 and MDA-MB-231 were purchased from ATCC (American Type Culture Collection, Manassas, VA, USA). Ambion’s Silencer select Pre-designed siRNA (Life Technologies**^®^**, Waltham, MA, USA) was used to silence the RBBP6 fragment. The pCMV6-AC-GFP mammalian expression vector (Blue Heron Company Los Angeles, CA, USA) was used to overexpress RBBP6. Overexpression was achieved by delivering RBBP6 transcript variant 3, which is the open reading frame (NM_032626.5), into the cell lines. Camptothecin (Calbiochem**^®^**, Berlin, Germany) and γ-aminobutyric acid (GABA) (Sigma-Aldrich, St Louis, MO, USA) were used as anticancer agents.

### Ethical statement

The study was approved by the North-West University Human and Health Ethics Committee in 2017. All patients gave written informed consent to the study.

### Cell culture

Human monolayer carcinoma and normal cell lines were cultured in HyClone**^®^** high glucose growth medium (DMEM) which was supplemented with 10% FBS and 1% antibiotic (penicillin/streptomycin) and routinely maintained at 37°C, with 95% humidity, in a 5% CO_2_ incubator. The cells growth media were replaced every second day of the week. The cells were sub-cultured when near-confluency and other subcultures were further preserved at ≥−80°C in a DMEM growth medium supplemented with 10% FBS and 20% dimethyl sulfoxide (DMSO).

### xCELLigence assay

For this assay, 1×10^5^ cells transfected with siRBBP6 and pRBBP6 were seeded in each well of the E-plates which had their background readings recorded by imposing the plates containing antibiotic-free medium to xCELLigence current flow. The E-plates were connected to the current flow supplied by the xCELLigence machine that was placed in a 37°C incubator for 24 hours before further treatment with GABA and camptothecin to allow cell attachment and to monitor cell growth. Posttreatment with the apoptosis-inducing agents, the E-plate was further incubated for a period of 48 hours. Cell index values were recorded at equal intervals.

### Immunohistochemistry

Immunohistochemical staining for RBBP6 proteins was performed in order to determine their expression and localization. Briefly, tissue sections were mounted on slides, dewaxed and rehydrated in 100% ethanol. The slides were then submerged in 100% absolute methanol containing 3% H_2_O_2_ for 20 minutes at room temperature (RT), and antigen retrieval was performed by heating the slides in 0.1 M sodium citrate (pH 6.0) at 80°C for 5 minutes. The slides were then allowed to cool to RT, and were washed in phosphate buffered saline Tween/tris buffered saline with Tween containing 1% BSA and incubated with specific primary antibodies for 18 hours after blocking for endogenous biotin and nonspecific binding. Slides were then washed with phosphate buffered saline Tween and incubated with secondary biotinylated antibody followed by streptavidin-peroxidase (LSAB kit; Dako, Woodstock, MA, USA). Visualization was done following incubation with 3,3’-Diaminobenzidine (Dako) and AEC (Dako). Slides were counterstained with Meyer’s hematoxylin, dehydrated and mounted with Depex followed by imaging with conventional light microscopy.

### Western blot analysis

Protein was extracted from a whole cell extract using RIPA buffer (1% NP-40 [Nonidet P-40], 0.5% sodium deoxycholate, 10% SDS, 3 µL/mL aprotinin and 5 µg/mL leupeptin in PBS; pH 7.4). Cells were washed twice with cold PBS and then resuspended in 500 µL RIPA buffer and collected by scraping 72 hours posttransfection and co-treatment with either staurosporine or camptothecin. The total protein was then separated from cell debris by centrifugation at 14,000 rpm for 15 minutes and quantified with Pierce**^®^** BCA Protein Assay Kit. The protein was heated at 95°C for 5 minutes and 30 µg was loaded per well for electrophoretic separation in 40% acrylamide gel preparation at 100 V for 1 hour. The protein was transferred onto a nitrocellulose membrane using the wet electro-transfer method overnight at 30 V followed by incubation with primary antibody.

### Annexin V/PI apoptosis assay

The type of cell death induced by the co-treatments was determined using a technique which employs Annexin V and PI. Annexin V detects the translocated or externalized phosphatidylserine (PS) residues from the inner to the surface of the plasma membrane and PI detects the cells with permeabilized plasma membrane. Briefly, cells were seeded in six-well plates and were transfected with siRBBP6 or pRBBP6 for 24 hours, which was followed by treatment with 100 µM GABA and 0.25 µM camptothecin and incubation for additional 24 hours. Following treatment, the cells were collected and washed with cold PBS, and then trypsinized and transferred to 15 mL tubes and were centrifuged at 1500 rpm for 2 minutes. The pellet (1×10^4^ cells/mL) was resuspended in 100 µL 1X binding buffer (Annexin V-FITC Apoptosis Detection Kit; Abcam**^®^**), and was incubated with 5 µL of Annexin V-FITC (Abcam) and 5 µL PI for 15 minutes at RT in the dark. After incubation, 400 µL 1X binding buffer was added and the cells were analyzed by flow cytometry within 1 hour.

### Confocal microscopy

In order to determine the morphological changes of the cells after co-treatments, a fluorescence confocal microscopy was employed. Briefly, cells treated with siRBBP6 and pRBBP6 were grown on coverslips mounted into six-well plates and were incubated overnight. This was followed by treatment with 0.25 µM camptothecin or 100 µM GABA, and incubation for further 24 hours. The cells were then washed with cold PBS, and stained with Annexin V-FITC/DAPI followed by fixation with 4% formaldehyde for detection of apoptosis. Furthermore, for protein expression analysis, 0.01% Triton X-100 was added to fixed cells and then immunostained with anti-GFP primary antibody after the cells were washed with 1% BSA blocking buffer. The cells were then washed with cold PBS followed by incubation with GFP-tagged secondary antibody and were imaged at 10× magnification using Olympus BX63 confocal fluorescence microscope.

### Caspase 3/7 activity

Apoptosis was quantified using caspase 3/7 activity assay, by measuring light intensity using Caspase-Glo**^®^** 3/7 (Promega Madison, WI, USA) according to the manufacturer’s specifications. Briefly, 5×10^4^ cells/well were seeded on a white 96-well microplate and subsequently treated with either siRBBP6 or pRBBP6 (expression vector) and were allowed to attach overnight, which was followed by treatment with either camptothecin or GABA for 24 hours. Then, Caspase-Glo reagent was added and incubated for 30 minutes at RT. The caspase activity was read using the Glomax**^®^**-96 microplate illuminometer and the results were presented as mean of relative light units (RLU).

### Real-time PCR

The Luminaris Color HI Green qPCR Master Mix (inqaba biotec, Pretoria, South Africa) dye was used for detection and quantification of the amount of gene expression of target cDNA. Briefly, cells were transfected with 30 nM siRBBP6 and 1 µg pRBBP6 and incubated for 48 hours followed by treatment with either 0.25 µM camptothecin or 100 µM GABA. After treatment, total RNA was isolated using the Quick-RNA™ Miniprep (Zymoresearch, Irvine, CA, USA) and was quantified using a Nanodrop spectrophotometer. Normalized amounts of total RNA were reverse transcribed using the ImProm-II Reverse Transcription System (Pro-mega) using manufacturer’s protocol. Concentrations of 2100 ng/µL cDNA were subjected to real-time quantitative PCR (qPCR) with the following specific primers under study: RBBP6, TP53, bax, bak, Bad, caspase-3, caspase-8 and Bcl-2. SYBR Green technology was used to quantify the results. The data were analyzed using REST 2009 software.

### Statistical analysis

The results of each set of experiments were expressed as mean±standard deviation. Levels of statistical significance were analyzed using paired Student’s *t*-test and *P*-values were considered statistically significant only when *P*<0.001, *P*<0.01 or *P*<0.05.

## Results

### The expression of RBBP6 in breast cancer tissue sections

**Figure 1:**
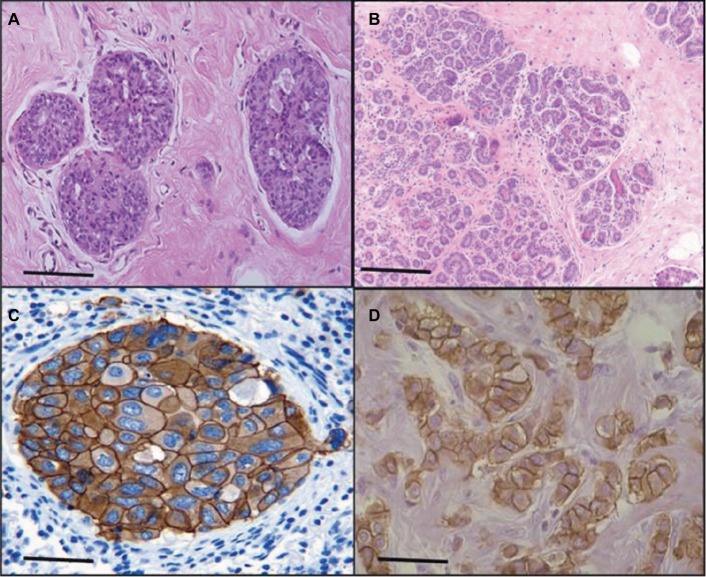
Expression of RBBP6 protein in subtypes of breast cancer. The micrograph shows normal breast tissue (**A**), a well-differentiated adenocarcinoma (**B**), tubular and papillary adenocarcinoma (**C**) and pulmonary adenocarcinoma (**D**). The bar magnification is 40×.

**Figure 2:**
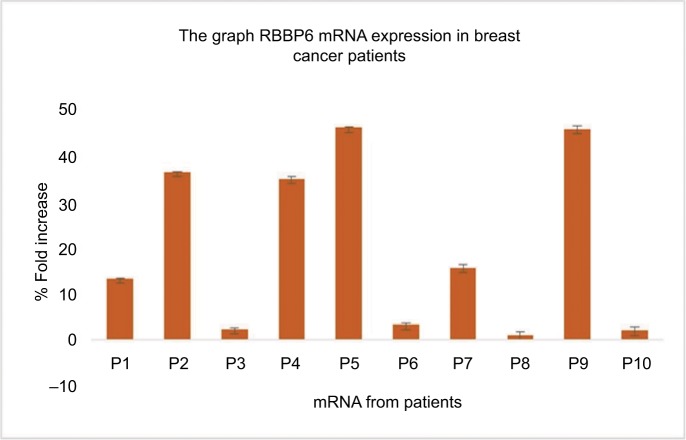
Quantitative PCR of RBBP6 mRNA levels in human breast cancer tissues. Samples were obtained from 10 breast cancer patients as indicated by P1–P10.

**Figure 3:**
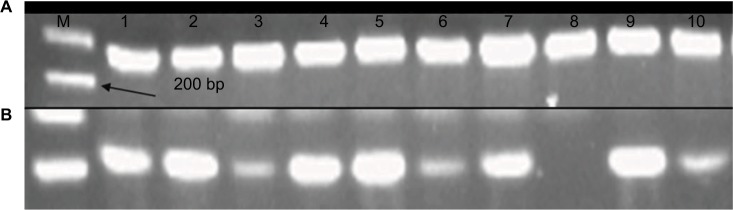
mRNA products of RBBP6 in the form of cDNA following qPCR were analyzed using agarose gel electrophoresis. Lane A indicates normalization using GAPDH primer, and lane B indicates qPCR products obtained from 10 tissue samples. **Abbreviations:** M, DNA ladder marker; qPCR, quantitative PCR.

The sections of breast cancer of the ductus and lumina showed strong cytoplasmic expression of RBBP6 protein, but weaker expression in the nucleus of tumor cells (Figure 1). The luminal contents also stained strongly for RBBP6. It is clear from micrographs in Figure 1C and D that the cytoplasmic localization and expression of RBBP6 protein were higher in the cancerous cells of glandular regions compared to grade II adenocarcinoma which showed moderate to less expression of RBBP6. The expression of RBBP6 was also stronger in advanced cancer tissues than in moderate one, suggesting that the more the cells divide the more they express RBBP6. At the gene level, it was clear from the band intensity observed in gel electrophoresis that the more advanced the cancer is, the higher the expression of RBBP6; however, at the earlier stages of the cancer, the expression is less (Figures 2 and 3). These results suggest the involvement of RBBP6 in cancer cell progression and therefore the protein could serve as a potential targeted therapeutic marker in breast cancer.

### RBBP6 gene silencing and protein overexpression in cell lines

**Figure 4:**
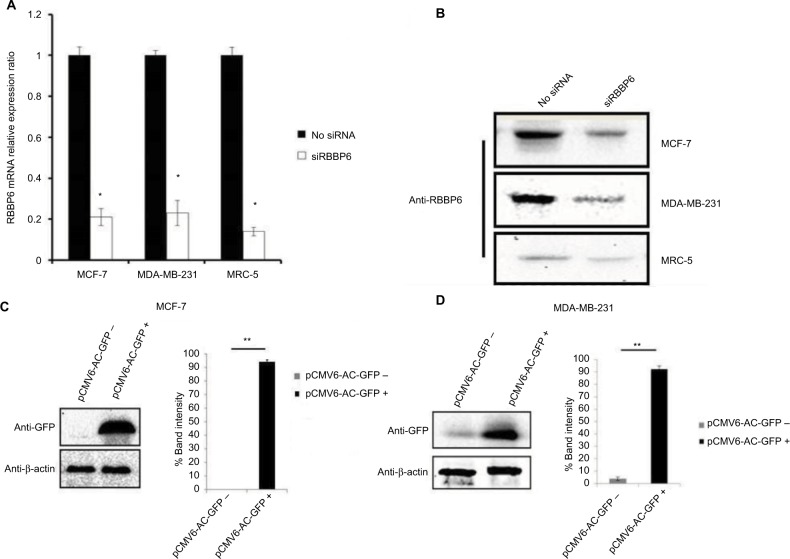
Transfection efficiency of RBBP6 gene silencing (**A** and **B**) and protein overexpression in MCF-7 (**C**) and MDA-MB-231 (**D**) cell lines. **Notes:** *P>0.05; **P>0.001.

To further understand the role of RBBP6 in cancer, both MCF7 and MDA-MB-231 cell lines were used to manipulate RBBP6 expression and measure its impact in cell proliferation. We used reverse transcription qPCR (RT-qPCR) to measure RBBP6 mRNA transcript levels following RNAi-based gene silencing as shown in Figure 4A. This technique resulted in a statistically significant downregulation of RBBP6 mRNA in both MCF-7 and MDA-MB-231 cell lines 48 hours post-transfection. There was approximately 79% and 77% gene silencing in both MCF-7 and MDA-MB-231, respectively, relative to non-transfected cells (Figure 4A). Furthermore, analysis of RBBP6 at a protein level using western blotting confirmed the observed gene silencing 72 hours posttransfection (Figure 4B). That is, RBBP6 protein expression was downregulated extensively following silencing in MCF-7 and MDA-MB-231 cell lines as shown on the electrophoretogram by the weaker band intensities as compared to non-transfected cell lysates (Figure 4B). In MRC-5 normal fibroblasts, RBBP6 expression was much lower prior to silencing, which was confirmed by the indistinct bands on the blot (Figure 4B). The open reading frame of RBBP6 transcript variant 3 (NM_032626.5) was delivered into cells (MCF-7 and MDA-MB-231; Figure 4C and D) by the pCMV6-AC-GFP expression vector. The GFP tag allowed positive identification of RBBP6 overexpression using anti-GFP primary antibody 72 hours posttransfection. As expected, transfection with pCMV6-AC-GFP led to about 90% GFP expression in both MCF-7 and MDA-MB-231 cells, which was directly proportional to RBBP6 protein overexpression (Figure 4C and D), whereas non-transfected cells showed no GFP expression (Figure 4C and D).

### Cell proliferation in response to RBBP6 silencing and overexpression

**Figure 5:**
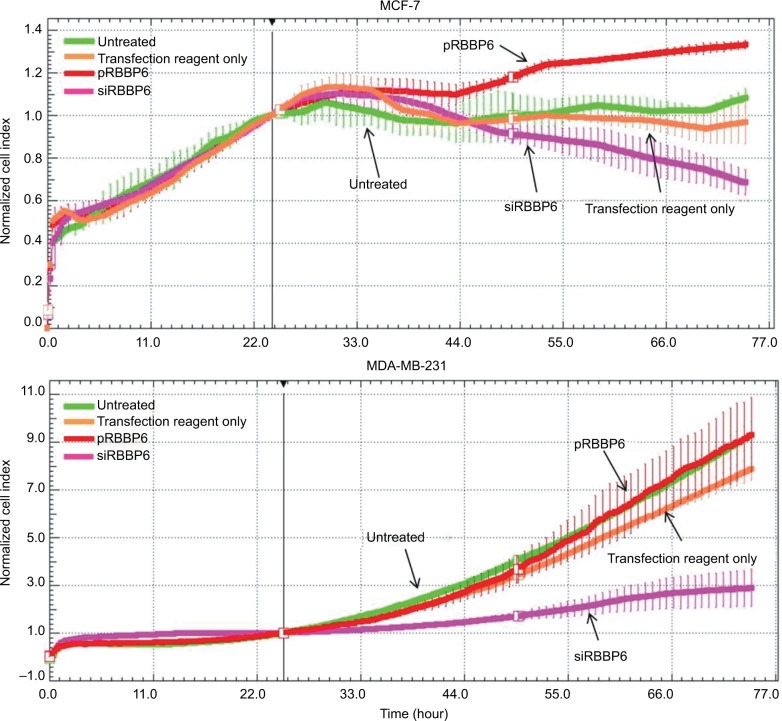
Growth of MCF-7 and MDA-MB-231 cells in the presence of siRBBP6 and pRBBP6 analyzed using the xCELLigence system.

Having successfully silenced and overexpressed RBBP6, we monitored cell proliferation in the presence of siRBBP6 and pRBBP6 transfections (Figure 5). While MDA-MB-231 cells generally showed slow response to siRBBP6 or pRBBP6, MCF-7 cell growth decreased with increasing exposure time to siRBBP6. Interestingly, the presence of pRBBP6 in MCF-7 cells seemed to favor cell growth more than in MDA-MB-231 cells (Figure 5).

### Apoptosis detection

**Figure 6:**
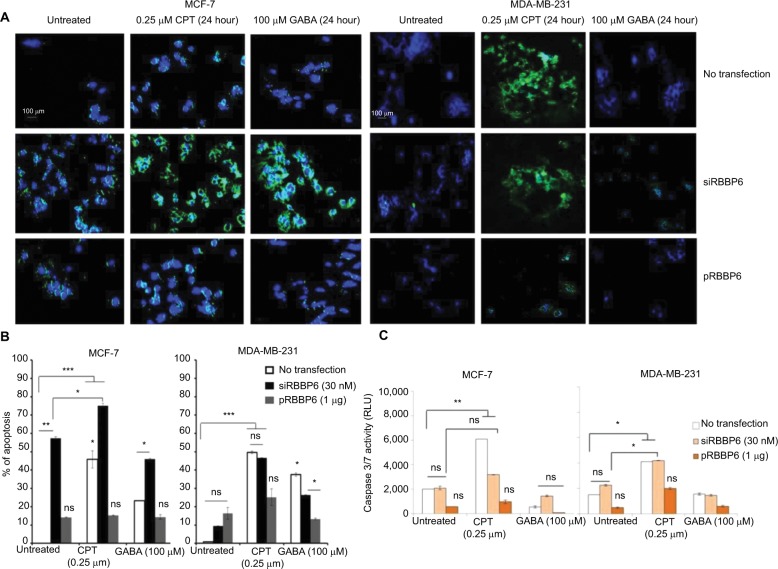
Analysis of apoptosis induction in MCF-7 and MDA-MB-231 cell lines following transfection of cells with siRBBP6 (30 nM) or pRBBP6 (1 µg) and co-treatment with camptothecin (0.25 µM) and γ-aminobutyric acid (100 µM) anticancer agents. Microscopic analysis of Annexin V/DAPI-stained cells (**A**). Statistical analysis of total apoptosis (early and late) following Annexin V/PI-based flow cytometry (**B**). Caspase 3/7 activity analysis by measuring a luminogenic product following cleavage of a caspase substrate by active caspases 3 and 7 present in the cell sample (**C**). **Notes:** *P<0.05; **P<0.001; ***P<0.0001. The images were captured at ×40. **Abbreviations:** CPT, camptothecin; GABA, γ-aminobutyric acid; ns, not significant; RLU, relative light units.

The abovementioned observations prompted us to analyze intracellular events that could have been triggered by RBBP6 gene silencing and overexpression thus affecting cell proliferation. First, we asked if the observed decrease in the cell growth of MCF-7 and MDA-MB-231 could be as a result of apoptosis induction (Figure 6). Fluorescence microscopy revealed a substantially positive Annexin staining only in MCF-7 cells transfected with siRBBP6 (Figure 6A). Although the Annexin V signal was weak in RBBP6-silenced MDA-MB-231 cells, co-treatment with the camptothecin anti-cancer agent led to a significantly strong signal in both cell lines, suggesting an apoptosis induction.

Morphological changes were observed in both the treated cells as compared to the untreated cells, and we discovered that treated cells undergoing apoptosis had adopted 11 irregular shapes (Figure 6) but untreated cells had their nuclei still intact and were round in shape (blue stain), whereas RBBP6 overexpression, on the other hand, resulted in little to no signal in both cell lines (Figure 6A). Similarly to GABA treatment alone, combination with siRBBP6 produced a weak fluorescence signal coupled with a loss of cell shape or with irregular cell shape of MDA-MB-231 cells (Figure 6). Co-treatment of pRBBP6 with either camptothecin or GABA failed to produce any visible green fluorescence signal in MDA-MB-231; however, cell shrinkage was especially observed in pRBBP6+GABA co-treatment (Figure 6).

Apoptosis was further confirmed using flow cytometry following transfection with siRBBP6 in both tumorigenic cell lines (Figure 6B). Although significant levels (57%) were detected in MCF-7, only 9% was observed in MDA-MB-231 cells following RBBP6 silencing (Figure 6B). Furthermore, exposure of RBBP6-silenced cells to camptothecin resulted in further increase of apoptosis, with MCF-7 cells showing up to 18% apoptosis increase, whereas MDA-MB-231 underwent a 37.6% increase. On the other hand, GABA co-treatment with siRBBP6 resulted in 29% apoptosis reduction in MCF-7 and 17% apoptosis increase in MDA-MB-231 when compared to RBBP6 silencing only. Transfection with pRBBP6 plasmid induced minimum amount of apoptosis across both cell lines, with 12% and 17% in MCF-7 and MDA-MB-231, respectively. Insignificant changes in apoptosis following co-treatment with pRBBP6+camptothecin and pRBBP6+GABA were observed in MCF-7 (15% and 13%, respectively) and MDA-MB-231 (27% and 15%, respectively) compared to pRBBP6-only transfection (Figure 6B).

Caspase 3/7 activity was not extensively changed after transfection with either siRBBP6 or pRBBP6 in comparison to untreated cells in both MCF-7 and MDA-MB-231 (Figure 6C). However, camptothecin increased the activity to 6000 RLU and 4000 RLU in MCF-7 and MDA-MB-231 cell lines, respectively. Co-treatment with siRBBP6 and camptothecin minimally sensitized caspase activity in MCF-7 cells (3800 RLU) and MDA-MB-231 cells (4100 RLU) in comparison to RBBP6 silencing only (Figure 6C). pRBBP6 alone or in combination with either camptothecin or GABA led to insignificant change in caspase 3/7 activity in both MCF-7 and MDA-MB-231 cells (Figure 6C).

### Quantification of genes involved in apoptosis

**Figure 7:**
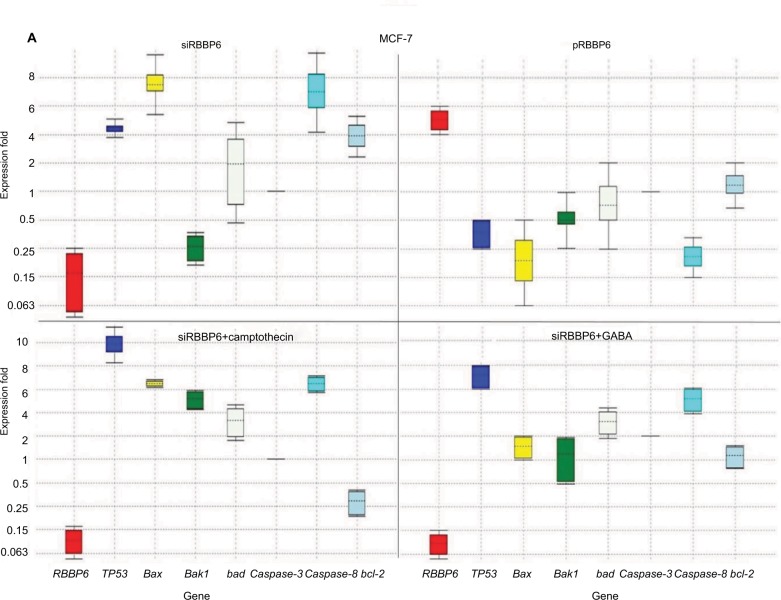

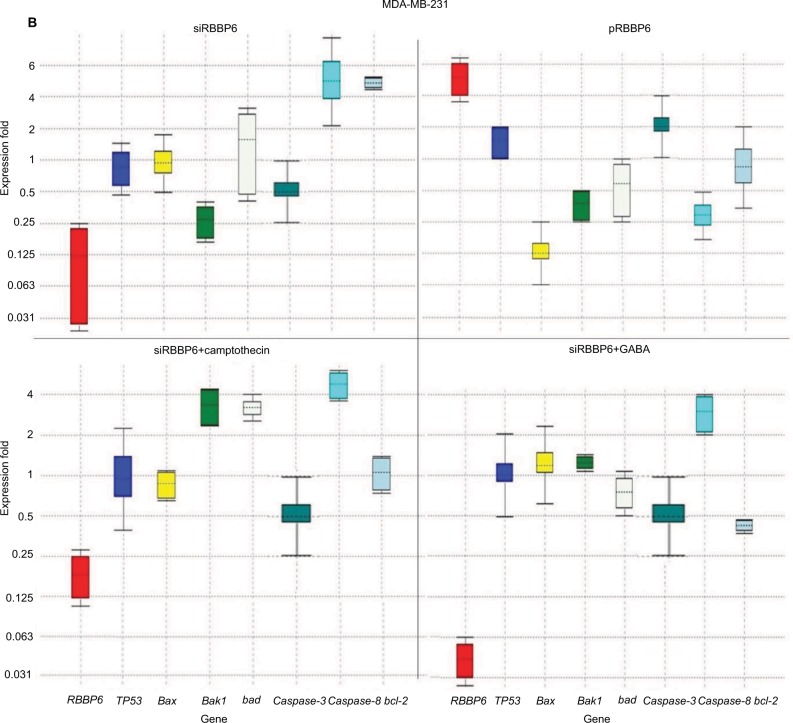
Relative quantification of gene expression in MCF-7 (**A**) and MD-MB-231 (**B**) cell lines performed using qPCR. Whisker box plots with expression ratio on the y-axis and gene type on the x-axis indicate quantification of mRNA isolated from cells treated with siRBBP6, pRBBP6, siRBBP6+camptothecin and siRBBP6+GABA. Gene expression is depicted by the color-coded whisker bars in the following manner: RBBP6 (red), TP53 (blue), bax (yellow), bak1 (light green), Bad (gray), caspase-3 (dark green), caspase-8 (cyan) and Bcl-2 (light blue). **Abbreviations:** qPCR, quantitative PCR; GABA, γ-aminobutyric acid.

RBBP6 downregulation (0.109) following silencing in MCF-7 led to TP53 upregulation by a mean factor of 4.61 (Figure 7A). No change was observed in Bad expression following RBBP6 silencing; however, Bcl-2 expression was increased by 3.8 and bax by 7.7 (Figure 7A). In contrast, silencing of RBBP6 led to a decrease in bak1 (0.25) and no change in caspase-3. RBBP6 upregulation (5.7) led to a decrease in TP53 (0.4), bak1 (0.5), Bad (0.75) and caspase-8 (0.18) and no change in caspase 3 (Figure 7A). RBBP6 silencing in combination with camptothecin led to downregulation of RBBP6 by 0.086, whereas TP53, bax, bak1, Bad and caspase-8 were upregulated by 10, 6.5, 5.8, 3.6 and 6.3, respectively. In contrast, the anti-apoptotic Bcl-2 was downregulated by a mean factor of 0.3 following siRBBP6+camptothecin treatment.

In combination with GABA, RBBP6 silencing (0.04) increased the expression of TP53 by 7.7 and caspase-8 by 5.3 (Figure 7A). In MDA-MB-231 cell line, RBBP6 silencing (0.08) had no effect on the expression of TP53, bax and Bad; however, bak1 and caspase-3 expression were downregulated by 0.25 and 0.5, respectively (Figure 7B), and caspase-8 and Bcl-2 were upregulated by 5.52 and 5.4, respectively (Figure 7B). Overexpression of RBBP6 (5.7) had no signifi-cant effect on TP53 expression, upregulated caspase-3 (2.00) and downregulated bax, bak1, Bad, caspase-8 and Bcl-2 by 0.125, 0.35, 0.56, 0.29 and 0.75, respectively (Figure 7B). Combination of RBBP6 silencing with camptothecin led to a decrease in RBBP6 expression by a mean factor of 0.17 and had no significant effect on TP53, bax, caspase-3 and Bcl-2 gene expression. Bak1, Bad and caspase-8 gene expression was increased by 3.21, 3.18 and 4.7, respectively, in response to siRBBP6+camptothecin co-treatment (Figure 7B). Co-treatment with siRBBP6+GABA reduced the expression of RBBP6 by a mean factor of 0.04; however, the change in expression of TP53, bax, caspase-3 and Bad was insignificant. Bak1 and caspase-8 genes were upregulated by 1.23 and 2.83, respectively; however, Bcl-2 was downregulated by 0.42 (Figure 7B).

## Discussion

Heterogeneity in breast cancer is still a major cause of drug resistance.^4^ In most cases, this type of resistance is characterized by either mutations in or inactivation of p53. Targeted therapy in combination with anticancer agents has shown to improve efficacy and side effects in this disease.^4^ In this study, we show through silencing and overexpressing RBBP6 in mt p53 and wt p53 breast cancer cell lines that restoring the functions of wt p53 sensitized cancer cells to anticancer agent through the activation of apoptosis machinery. The expression of p53 is what sets the two cell lines apart, with MCF-7 being shown to generally express wt p53 and MDA-MB-231 expressing mt p53.^8^ From the initial studies, we evaluated the expressional pattern of RBBP6 in cancer tissues and, as indicated in Figures 1 and 2, RBBP6 was variably expressed depending on the advancement of the cancer, with more advanced cancer expressing high levels of RBBP6 and moderate cancers expressing low levels of RBBP6.

RBBP6 was observed to be concentrated or overexpressed in the cytoplasm and partially in the nuclei in moderate cancers (Figure 1). Similar results were reported by Motadi et al^12^ in which RBBP6 was highly expressed in stage IV lung cancer, while in stages II and I, lower expression was observed. Further studies were reported in cervical and colorectal cancers with RBBP6 overexpressed in well-differentiated cancers. The fact that RBBP6 was found to be expressed differentially in several tissues suggests a pivotal role it plays in cancer progression and as a potential biomarker for targeted cancer therapy. From our initial gene silencing and overexpression studies, it was observed that RBBP6 was knockdown by close to 90% of its initial expression in all cells as shown by both qPCR and Western blot. This successful silencing is credited to the high specificity elicited by the synthetic short interfering RNA molecules in directing homology-dependent control of gene activity using the cell’s own machinery.^9^,^10^ In terms of RBBP6 overexpression, we have targeted the isoform 3 which can be detected by all the RBBP6 isoforms and it is suggested to be the regulatory domain of the entire genes. From our studies, we have seen a successful expression of RBBP6 both at mRNA and protein levels, which was confirmed by the expression of the GFP reporter. The mechanism by which RBBP6 promotes cell proliferation is still unclear. However, the theory that it stabilizes chromosomal fragile sites during DNA replication might account for its proliferative function.^11^ Another possible explanation could be that RBBP6 negative regulation of wt p53 inhibits cell cycle leading to increased cell progression.^12^ This might also help to explain the different responses observed in the two cell lines where the mt p53-expressing MDA-MB-231 appeared to be less responsive to RBBP6 silencing compared to MCF-7. From these observations, it was important to us to quantify the expression of p53 following silencing and overexpression. From our observed results, there was over fourfold increment in p53 expression associated with siRBBP6 in MCF7 but there was no significant change in MDA-MB-231. The opposite was true with RBBP6 overexpression which showed insignificant or slightly reduced levels of p53. The results suggest the role of RBBP6 in p53 expression and activation. p53 is the genomic guardian of the cell apoptosis in the guardian machinery of cell survival or death and plays a crucial role in deciding whether cells progress through cell cycle even if damaged. The defects in this process have been reported by many researchers^13^ as the contributing factor to cell apoptosis. Following earlier interesting results on p53 expression levels, it was important for us to evaluate the deeper molecular impact of its expression or restoration. We evaluated apoptosis induction using both flow cytometer and microscopy. As extensively reported, the hallmarks of apoptosis associated with microscopy are cell shrinkage and blebs while other stain-dependent characteristics include PS externalization.^14^ Highly significant apoptosis induction was observed in MCF-7 cell line, whereas in MDA-MB-231 cells, there was insignificant apoptosis detected following RBBP6 silencing (Figure 6). In RBBP6 overexpression, no cell line elicited significant levels of apoptosis induction. These observations might suggest that RBBP6 silencing which resulted in p53 accumulation in MCF-7 was responsible for the reported cell death by apoptosis as seen in this study. p53-mediated apoptosis occurs via the intrinsic pathway where p53 transactivates and promotes translocation of bax to the outer mitochondrial membrane, thereby oligomerizing with itself or bak1 in order to porate the membrane.^14^–^16^ Porous mitochondrial membrane allows the release of cytochrome c and other factors responsible for inducing apoptosis.^17^–^19^ Upregulation of bax seen in MCF-7 might therefore be as a result of p53 activation, thus confirming the observed induced apoptosis through the mitochondrial pathway. Even though caspase 3/7 activity was not significant in MCF-7, probably due to the fact that the cell line is caspase-3-deficient, from all the observed results, it was clear that RBBP6 led to apoptosis induction that was associated with p53 restoration in wt p53-expressing cells while mt p53 cells remained progressive. In recent years, combinational therapy is the most recommended form of cancer-combating mechanism. Combinational therapy identifies the molecular effect in cancer cells and replaces it, followed by chemotherapy or any appropriate therapy resulting in much improved and side-effect-free treatment.^12^ We then went on to exploit the combinational mixture of RBBP6-silencing and apoptosis-inducing anticancer agents, camptothecin and GABA, to verify if our hypothesis might be true for MDA-MB-231 since they failed to elicit significant levels of apoptosis induction when treated with siRBBP6 only. Camptothecin is a naturally occurring compound that was extracted from a Chinese medicinal plant and has since been shown to have anticancer effect.^20^ GABA, the inhibitory neurotransmitter that is found in the central nervous system of vertebrates,^21^,^22^ was chosen as an anticancer agent in this study due to its ability to induce apoptosis, cell cycle arrest and blockade of tumor cell migration.^21^,^23^ Similarly to our previously reported results in MCF-7, camptothecin sensitized MCF-7 cells to apoptosis. Interestingly, camptothecin in combination with siRBBP6 induced apoptosis in MDA-MB-231 cells, although the effect was not synergistic as was with MCF-7 cells. GABA did not enhance apoptosis in MDA-MB-231 cell line whereas it did in MCF-7 cells. RBBP6 overexpression, on the other hand, did not induce or sensitize significant apoptosis in both cell lines. Besides the genotypic nature of p53 expression in these two cell lines, the mechanisms of action underlying the two anticancer agents might account for the differences observed in apoptosis levels. Camptothecin induces apoptosis by inhibiting topoisomerase I during the release of supercoils in DNA replication by binding to the single-strand nicks created by the enzyme, thus preventing religation. This leads to damaged DNA which then triggers the transactivation of p53 and a subsequent apoptosis induction.^20^,^24^,^25^ Therefore, this might account for the synergistic effect observed between camptothecin and RBBP6 silencing in inducing apoptosis since the mechanisms of action for both strategies seem to reactivate the function of p53. No distinctive mechanism of apoptosis induction has been described for GABA yet; however, its affinity for G-protein-coupled receptors has been shown to activate adenylyl cyclase which in turn is responsible for activating PKA/cAMP signaling pathway which is responsible for phosphorylating several target proteins including those that lead to the induction of apoptosis.^21^,^23^ Several studies have been done in other cancers where GABA was shown to induce apoptosis.^21^,^23^,^26^,^27^ In the context of our study, apoptosis induction in response to GABA was observed as well, however at minimal levels in both cell lines. The reason for this is not known; however, we suspect that the anticancer effects of GABA might be highly effective against cell migration as opposed to apoptosis. This is because several studies have proven, especially in liver cancer, that the effect of GABA is associated with cell migration.^22^,^28^ Indeed, breast cancer cell lines, MDA-MB-231 and MCF-7, show differential susceptibilities in apoptosis induced by RBBP6 silencing alone or in combination with either camptothecin or GABA. This might be as a result of the distinct p53 statuses of the two cell line models. The p53 tumor suppressor carries missense mutations in most human cancers, and are generally more stable and highly expressed than wt p53.^29^ This is not the case with the other two well-studied tumor suppressors Rb and APC as they have been shown to carry deletion and truncation mutations.^29^ Missense mutations are often associated with gain-of-function phenotypes; mt p53 in MDA-MB-231 has been reported to have the ability to interact with certain peptides in order to prevent apoptosis induction. One example of such peptides is called phospholipase D (PLD) and has been shown to be overexpressed in MDA-MB-231 cell line where it interacts with mt p53 to generate survival signals that suppress DNA damage-induced apoptosis.^21^,^23^,^30^ This interaction might therefore be responsible for the restricted apoptosis induction observed in MDA-MB-231 in response to silencing and co-treatment, especially in camptothecin co-treatments which induce apoptosis by damaging the DNA. Successful silencing and overexpression of RBBP6 have enabled the understanding of the effects of RBBP6 on both breast cancer cell lines. We have reported that both the breast cancer cell lines, MCF-7 and MDA-MB-231, respond to RBBP6 silencing by differentially ceasing to grow, whereas overexpression of RBBP6 maintains their increase in proliferation. Furthermore, MDA-MB-231 cell line seemed to respond to RBBP6 silencing at a slow rate as opposed to MCF-7 cells. This pattern appeared to be repetitive in apoptosis detection since MDA-MB-231 cells showed a weak response whereas MCF-7 cells showed a significant amount of apoptosis induction. This apoptosis was further confirmed by expression of pro-apoptotic genes. These differential characteristics elicited by the two cell lines were coined on their p53 statuses, wherein wt p53 accumulation in MCF-7 was associated with apoptosis induction especially because bax pro-apoptotic gene was upregulated. mt p53 in MDA-MB-231 is suspected to have had interactions with the pro-survival PLD peptide, thus preventing apoptosis induction in this cell line. All the results, together with the ones observed in tissue section, point to a correlation in RBBP6 expression and cell proliferation.

## Conclusion

It is evident that the genotypic status of p53 in MCF-7 and MDA-MB-231 breast cancer cell lines plays a critical role in the way they respond to RBBP6-targeting treatment and/or co-treatment with apoptosis-inducing therapeutic agents.

Furthermore, we have demonstrated that the ability of these cells to undergo apoptosis in the absence of RBBP6 is partly as a result of p53 reactivation, which demonstrates a clear indication of the initial interaction between p53 and RBBP6 in breast cancer. From tissue sections, we further showed that RBBP6 expression is associated with advancement of cancer. These observations further highlight the significance of understanding the different subtypes of breast cancer as far as treatment is concerned because the heterogeneous nature of this disease requires patient-specific therapies, thus making it difficult to manage breast cancer.
